# Deregulated Long Non-Coding RNAs (lncRNA) as Promising Biomarkers in Hidradenitis Suppurativa

**DOI:** 10.3390/jcm13103016

**Published:** 2024-05-20

**Authors:** Uppala Radhakrishna, Uppala Ratnamala, Devendrasinh D. Jhala, Lavanya V. Uppala, Aaren Vedangi, Nazia Saiyed, Maulikkumar Patel, Sushma R. Shah, Rakesh M. Rawal, Gregor B. E. Jemec, Tommaso Mazza, Gianluigi Mazzoccoli, Giovanni Damiani

**Affiliations:** 1 Department of Anesthesiology and Perioperative Medicine, University of Pittsburgh, Pittsburgh, PA 15261, USA; 2 Department of Life Sciences, School of Sciences, Gujarat University, Ahmedabad 380009, Indiarakeshmrawal@gmail.com (R.M.R.); 3 Department of Zoology, School of Sciences, Gujarat University, Ahmedabad 380009, India; 4 Peter Kiewit Institute, College of Information Science & Technology, The University of Nebraska at Omaha, Omaha, NE 68182, USA; 5 Department of Clinical Research, KIMS ICON Hospital, ICON Krishi Institute Medical Sciences, Sheelanagar, Visakhapatnam 530012, India; 6 Department of Obstetrics and Gynecology, Corewell Health William Beaumont University Hospital, Royal Oak, MI 48073, USA; 7 Bioinformatics Unit, Gujarat University, Ahmedabad 380009, India; maulik.patel001@gmail.com; 8 Department of Obstetrics and Gynecology, BJ Medical College Institute of Medical Post-Graduate Studies and Research, Ahmedabad 380016, India; 9 Department of Dermatology, Zealand University Hospital, 4000 Roskilde, Denmark; gbj@regionsjaelland.dk; 10 Bioinformatics Unit, IRCCS “Casa Sollievo della Sofferenza”, Opera di Padre Pio da Pietrelcina, Cappuccini Avenue, 71013 San Giovanni Rotondo, Italy; 11 Division of Internal Medicine and Chronobiology Laboratory, Department of Medical Sciences, IRCCS “Casa Sollievo della Sofferenza”, Opera di Padre Pio da Pietrelcina, 71013 San Giovanni Rotondo, Italy; 12 Department of Biomedical, Surgical and Dental Sciences, University of Milan, 20122 Milan, Italy; 13 Italian Center of Precision Medicine and Chronic Inflammation, University of Milan, 20122 Milan, Italy; 14 Fondazione IRCCS Ca’ Granda, Ospedale maggiore Policlinico, 20122 Milan, Italy

**Keywords:** epigenetics, Hidradenitis suppurativa, biomarkers, chronic skin disorders, bioinformatics, long non-coding RNA, precision medicine

## Abstract

**Background/Objectives:** In recent times, epigenetics alterations in Hidradenitis suppurativa (HS) have been explored and exploited translationally to guide investigation of new therapeutic approaches. On the other hand, long noncoding RNAs (LncRNAs), main regulators of the epigenetic status of the human genome, have been scarcely investigated, notwithstanding their potential relevance in broad pathogenesis comprehension. Here, we aim to explore the methylation pattern of lncRNAs in HS. **Methods**: In this case-control study, 24 HS patients and age-, sex- and BMI-matched controls were analyzed to characterize the methylome of lncRNA genes in peripheral blood cells. Gene ontology analysis (GO), Kyoto Encyclopedia of Genes and Genomes (KEGG) pathway analysis, protein–protein interaction (PPI) network, and MCODE analysis were performed. **Results**: A set of fifteen lncRNA genes exhibited significantly differential methylation patterns, with ten of them showing hypomethylation and five displaying hypermethylation at specific CpG sites. The hypomethylated lncRNA genes were *DLEU2*, *MESTIT1*, *CASC2*, *TUG1*, *KCNQ1DN*, *PSORS1C3*, *PCA3*, *DSCR8*, *RFPL1S*, and *PVT1*, while the hypermethylated ones were *HAR1A*, *FAM66B*, *SNHG9*, *HCG9*, and *HCP5.* These lncRNA genes have been linked to various important biological processes, including cell proliferation, apoptosis, inflammation, chronic inflammatory skin diseases, and wound healing. Their altered methylation status suggests potential roles in regulating these processes, and may contribute to HS pathogenesis and healing mechanisms. **Conclusions**: This study revealed an interesting dysregulation pattern of definite lncRNAs in the methylome which is linked to both the development of HS and its comorbidities. Epigenetically altered lncRNAs genes could represent useful biomarkers, and could help in guiding innovative treatment strategies.

## 1. Introduction

Hidradenitis suppurativa (HS), also known as *acne inversa* or Verneuil’s disease, is a chronic and recurrent inflammatory dermatosis of the hair follicle with severe negative impact on quality of life and associated with important co-morbidities. HS lesions most commonly occur in intertriginous areas and areas rich in apocrine glands. Among the most common are the axillary, groin, perianal, perineal, and inframammary locations. Because of the associated pain, sensitive locations, drainage, odor, and scarring, this condition may have a negative psychosocial impact [1]. Numerous definite genetic changes [2,3] and epigenetic modifications [4,5,6] are associated with HS susceptibility, disease onset/progression and response to treatment. Current basic science research focusing on the etiology, pathophysiology, and treatment of HS is ever-increasing [7]; however, it remains elusive and poorly informative as regards targeted therapies [8,9,10]. Furthermore, while exposure to a number of environmental factors is capable of modulating disease severity, flares, and even drug response, the pathogenesis of disease outcomes modifications caused by ambient components remains unclear. The framework of disease mechanisms driving HS may involve long noncoding RNAs (lncRNAs), which are transcripts longer than 200 nucleotides in length that lack protein-coding capacity [11]. LncRNA biogenesis is related to specific subcellular localizations and functions; through definite interactions with DNA, RNA and proteins can control chromatin function, regulate the assembly and function of membraneless nuclear bodies, modify cytoplasmic mRNA stability and translation, and affect intracellular signalling pathways, in due course impacting gene expression in various biological and pathological settings [12]. 

Furthermore, lncRNAs fine-tune numerous cellular processes such as splicing, the cell cycle, apoptosis, pluripotency preservation, embryonic development, and cell differentiation [13,14]. lncRNAs are transcribed from different genomic regions, such as exons, promoters, and intergenic regions, and are important regulators of epigenetic status in the human genome, influencing gene expression [15]; according to Gencode Release v43, (https://www.gencodegenes.org, accessed on 23 March 2023), 19,928 lncRNA genes produce 25,407 lncRNA transcripts, and the number is growing steadily. LncRNAs are uniquely expressed in specific cell types to a greater degree than protein-coding RNAs (mRNAs), and are regarded as crucial in disease onset, showing specific expression in different cancer types. They are considered valuable biomarkers supportive of diagnosis. Furthermore, lncRNAs are enriched at numerous imprinted gene clusters and participate in genomic imprinting, a multifaceted and highly controlled process leading to the monoallelic silencing of definite genes based on the parent-of-origin of the allele imprinting processes [16]. 

Clinically, lncRNAs play a role in the pathogenesis of various diseases, including cardiovascular disease, atherosclerosis, Alzheimer’s disease [17], dyslipidemia, and metabolic syndrome [18]. lncRNAs represent valuable biomarkers and druggable targets depending on tissue-specific and condition-specific expression patterns. 

Recently, lncRNAs have gained attention for their potential roles in modulating keratinocyte differentiation and inflammation [19]. Deregulated lncRNAs have been implicated in aberrant keratinocyte differentiation and disruption of epidermal homeostasis, and are crucially involved in the pathogenesis of several hyperproliferative skin diseases, including psoriasis, hypertrophic scars, cutaneous squamous cancer, melanoma and haemangiomas [20]. Recent studies have shown that lncRNAs play important roles in epidermal development, keratinocyte differentiation, and melanocyte function [21]. Notwithstanding this, the role played by lncRNAs in HS onset and progression is not adequately yet clear. 

The aim of this study was to explore the outline of DNA methylation at lncRNA coding genes in the peripheral blood of HS patients and age-matched controls through genome-wide analysis of DNA methylation patterns. 

## 2. Materials and Methods

### 2.1. Study Design

This study was approved by the Institutional Review Board of Beaumont Health System, Royal Oak, MI, USA (HIC#: 2015-172, 21 May 2015). The study followed ethical guidelines by obtaining written informed consent from all participating individuals, adhering to the principles outlined in the Helsinki Declaration. To ensure reliable and meaningful results, individuals diagnosed with HS were carefully paired with healthy controls matched based on similar characteristics such as age, gender, and body mass index (BMI). The demographic and clinical characteristics of patients and controls were previously reported [6].

### 2.2. Hidradenitis Suppurativa Sample Selection and Statistical Methods

Three independent board-certified dermatologists (RR, DGS, TM) at VS Hospital, Ahmedabad, India, employed a visual-aided questionnaire to conduct a thorough self-assessment of HS in patients. This questionnaire was specifically designed to aid in the evaluation of HS-related symptoms and their severity [22]. The diagnostic process and sample assessment were carried out according to the guidelines from the European Hidradenitis Suppurative Foundation (EHSF) [23]. 

The severity of the disease was assessed through grading system widely used to typify the extent of disease in HS patients and the impact of the condition on the affected individuals, including the Hurley score [24], HS Severity Score System (IHS4) [25], and Autoinflammatory Disease Damage index (ADDI) [26]. 

Inclusion criteria were (a) adult patients (>20 years of age) with a diagnosis of HS with a duration greater than 5 years, (b) at least a Hurley stage II severity, (c) IHS4 > 3 points, (d) ADDI < 3 points, (e) newly diagnosed HS (<3 months), and (f) untreated for at least 6 months. 

Exclusion criteria were: (a) syndromic HS as defined by Van der Zee and Jemec clinical phenotypes [27], (b) smoking, (c) fasting regimens [28] and/or particular diet regimens different from omnivore, (d) alcohol abuse (Alcohol Use Disorders Identification Test (AUDIT) > 7 points), (e) drug addiction, (f) use of concomitant medications, including contraceptives and enzyme-inducing foods (i.e., grapefruit) (g) treated for HS, (h) chronic inflammatory/infectious diseases or history of cancer in the previous 5 years, and (m) persons unable to provide informed consent for any reason.

### 2.3. Statistics and Bioinformtic Analyes

Data (IDAT files) were normalized using Genome Studio 2.0 software (Illumina Inc., San Diego, CA, USA, accessed on 2 April 2024) functional normalization and determined Cytosine methylation levels (ß-value) for each CpG site. Before analysis, we removed all CpG-probes that had missing ß-values. Differential methylation was assessed by comparing the ß-values for cytosine at each CpG locus in HS versus controls. To avoid confounding factors, we removed probes associated with sex chromosomes, non-specific probes, and probes targeting CpG sites within 10 bp of SNPs (each of which listed dbSNP entries within 10 bp of the CpG site). Further, SNPs with a minor allele frequency (≤0.05) were only considered for forwarding analysis. Significantly differently methylated CpG sites between HS and controls were defined based on preset cutoff criteria (FDR *p* < 0.05). Multiple CpG sites within a gene were resolved by selecting the CpG with the highest AUC ROC ranking and the lowest *p*-value. The *p*-value for methylation differences between case and control groups at each locus was calculated as previously described [6]. Raw and FDR *p*-values corrected for multiple testing (Benjamini–Hochberg test) were calculated. The AUC for combinations of loci was calculated using the ‘R’ program ‘ROCR’ package (v3.5.0) (accessed on 2 April 2024).

### 2.4. DNA Preparation and Methylation Analysis

We collected whole blood samples from 24 individuals diagnosed with HS and 24 healthy individuals as controls. Genomic DNA was extracted from these blood samples using the Gentra Puregene^®^ Blood Kit (Qiagen, Venlo, The Netherlands). To analyze DNA methylation patterns, the extracted DNA was subjected to a sodium bisulfite conversion process. This conversion process was carried out following the manufacturer’s protocol, which involved using the EZ 96-DNA methylation kit (Zymo Research, Irvine, CA, USA). A comprehensive description of the methodology used for the entire process has been previously published in [4]. 

### 2.5. Heatmap

The ComplexHeatmap module (v1.6.0) in the R package (v3.2.2) was utilized to create a heatmap. The heatmap displayed the distribution of methylated CpG sites within the CYP coding regions, with each site representing an individual data point. The purpose of this analysis was to visualize the methylation patterns across these regions. We utilized Ward’s method to perform hierarchical cluster analysis on the samples [29]. For comparison between HS and controls, CpG sites with FDR *p*-values ≤ 0.05 were considered significantly differently methylated. The area under the receiver operating characteristic (AUC-ROC) was calculated based on methylation levels at the most significantly differently methylated CpG loci. 

### 2.6. Principal Component Analysis

Principal Component Analysis (PCA) is a powerful data transformation technique extensively employed for enhancing data visualization and performing feature extraction by reducing the dimensionality of the dataset. PCA serves as a valuable feature extraction tool. Used in various fields, including epigenetics, it allows the most important features that contribute the most to the overall variance between groups to be identified and selected. The R function “prcomp” was used to compute principal components (PCs), then PC1, PC2, and PC3 were used for the PCA distribution plot. The 3D PCA distribution plot was generated using the R package “ggplot2”.

### 2.7. Protein–Protein Interaction Network and MCODE Analysis

Based on a database search (http://bio-annotation.cn/lncrna2target/search.jsp, accessed on 2 April 2024) of all 15 lncRNAs, 38 mRNA transcripts were found to be their targets. These genes were further subjected to string protein–protein interaction network analysis to search for association between them. Protein–protein interaction mapping is the process of identifying the physical interactions between proteins in a biological system. The STRING database (https://string-db.org/, accessed on 2 April 2024) is a valuable resource for this purpose, as it provides information on known and predicted protein–protein interactions based on a variety of sources, including experimental data, co-expression patterns, and text mining.

In the present study, protein–protein interaction (PPI) mapping was performed using the STRING database for multi-protein options in Organism: *Homo sapiens*. A total of 38 target proteins retrieved from lncRNA targets were subjected to PPI. The interaction detection method was set to experimentally validated, co-expressed, and curated genes, with the confidence score threshold set to high (>0.7).

### 2.8. Protein–Protein Interaction Network and MCODE Analysis

During the initial phase of the study, a comprehensive list of genes of interest was meticulously compiled. After consolidating the data, the subsequent pivotal phase involved delving into the complexities of potential protein–protein interactions (PPIs) linked to these genes. 

To fulfill this objective, the renowned STRING database (version 12.0 https://string-db.org/, accessed on 2 April 2024) played an indispensable role. Given the immense amount of data present in the realm of biological interactions, it was essential to filter and streamline the data to our specific needs. Thus, a set of interaction sources was activated to uphold the quality and pertinence of the gathered data. These sources encompassed: text mining, which derives interactions from the vast repository of scholarly literature; experiments, spotlighting interactions identified in tangible experimental scenarios; databases, integrating insights from various curated biological databases; and co-expression, shedding light on genes or proteins that express concurrently. To further refine the interaction data, a high-confidence interaction score threshold was set at 0.700. This strategic filtration ensured that only the most credible interactions were considered. Subsequently, to manifest these interactions in a more visually coherent manner, the PPI network extracted from STRING was channeled into Cytoscape software (https://cytoscape.org, accessed on 2 April 2024). This platform was instrumental in providing a more tangible representation, facilitating a deeper understanding and analysis of the interaction dynamics.

### 2.9. Ingenuity Pathway Analysis

We performed Ingenuity Pathway Analysis (IPA QIAGEN, Aarhus, Denmark) to implement wide-ranging data analysis to recognise investigational outcomes, envisage downstream influences, and recognize novel targets/biomarkers in the setting of HS.

## 3. Results

### 3.1. Identification of Dysregulated CpGs in HS

Epigenome-wide DNA methylation profiling with the Illumina Epic array (Illumina Inc., San Diego, USA) was performed on blood DNA samples from 24 HS patients and 24 age- and sex-matched controls to explore changes in DNA methylation at lncRNA coding genes. We identified fifteen significantly differentially methylated lncRNA coding genes in blood, of which ten were hypomethylated (*DLEU2*, *MESTIT1*, *CASC2*, *TUG1*, *KCNQ1DN*, *PSORS1C3*, *PCA3*, *DSCR8*, *RFPL1S* and *PVT1*) and five were hypermethylated *HAR1A*, *FAM66B*, *SNHG9*, *HCG9*, and *HCP5*) at CpG sites (FDR *p*-values ≤ 0.05) associated with the fifteen genes. All dysmethylated CPGs are listed in Table 1. 

### 3.2. Validation

**Figure 1:**
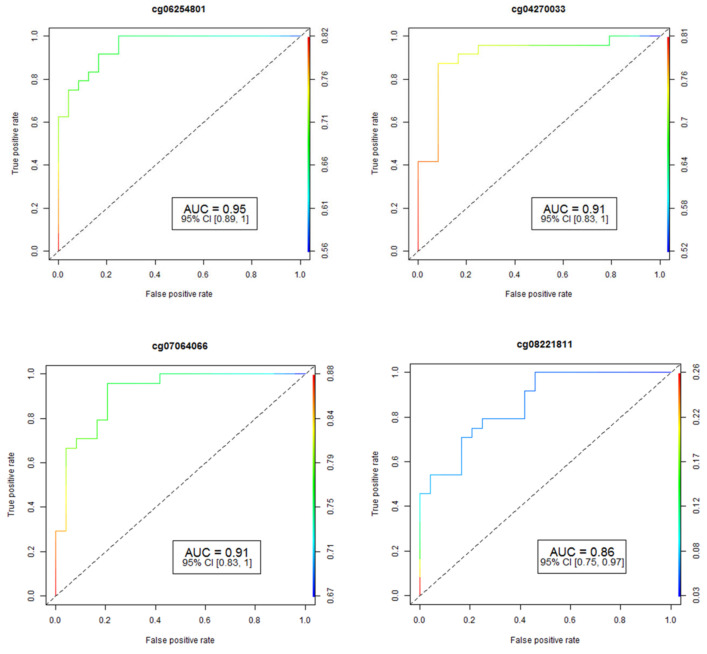
Analysis of receiver operating characteristic curves (ROC) based on AUC ROC and FDR *p*-values for four of the most significant CpGs associated with HS. The study identified fifteen differentially-methylated CpG sites in fifteen genes that have an area under the ROC curve ≥ 0.75 (*p*-value ≤ 0.05) for HS prediction. AUC: area under the receiver operating characteristics curve; 95% CI: 95% confidence interval. Lower and upper confidence intervals are given in parentheses.

The accuracy and reliability of the data concerning CpG methylation changes were rigorously assessed through pyrosequencing, a well-established method for validation. To determine the performance of individual CpG loci, we employed the Area Under the Receiver Operating Curve (AUC-ROC), a widely accepted metric in this field. The results for the four most promising CpG loci are visually represented in Figure 1, providing valuable insights into their predictive capabilities.

### 3.3. Evaluation of Heatmaps

**Figure 2:**
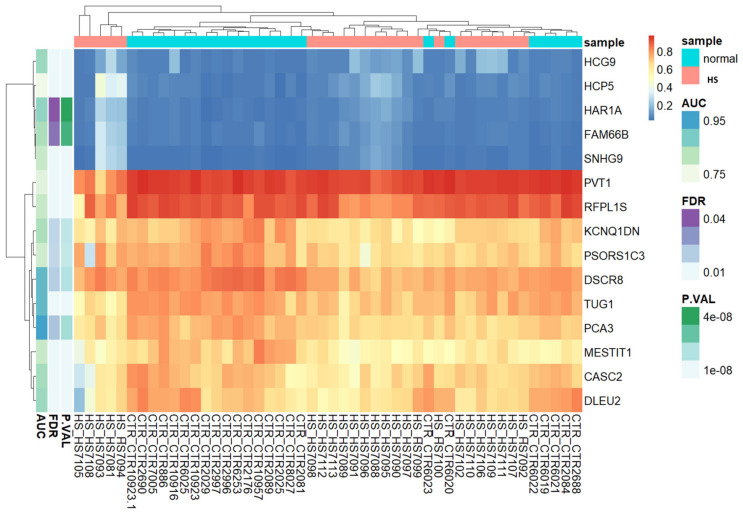
Unsupervised hierarchical clustering and heat map of the methylation data of fifteen CpGs with (Δβ) > 0.2 and FDR *p* values < 0.05 between HS cases and controls. The heat map colors correspond to lncRNA expression, as indicated in the color legend. The cases with HS phenotype are shown in red boxes and normal controls in green boxes. Blue and yellow indicate 0 and 1 methylation, respectively. The probes showing “hypermethylated” cases are represented by yellow vertical bars, and those showing “hypomethylated” cases by blue ones.

Based on lncRNA-related CpG methylation markers, the heatmap unequivocally demonstrates the presence of two discernible clusters of CpGs: one corresponding to HS patients, and another representing the control group (Figure 2). This compelling evidence supports the notion that these methylation markers serve as highly reliable indicators for distinguishing between HS-affected patients and unaffected individuals. In essence, our findings corroborate the accuracy and efficacy of these methylation markers for accurately discriminating between the two study groups.

### 3.4. PCA

**Figure 3:**
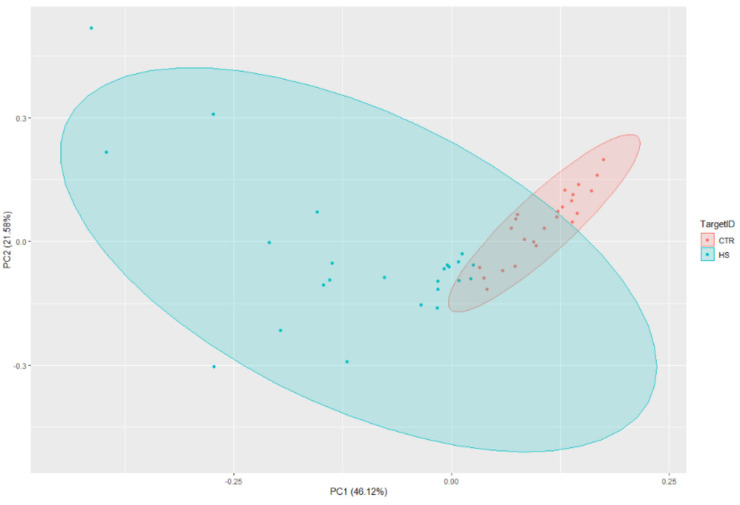
Principal Component Analysis (PCA) with lncRNA associated gene markers.

The PCA analysis provided strong evidence of distinct separation between the HS cases and controls (Figure 3).

### 3.5. Protein–Protein Interaction Network and Modular Analysis

**Figure 4:**
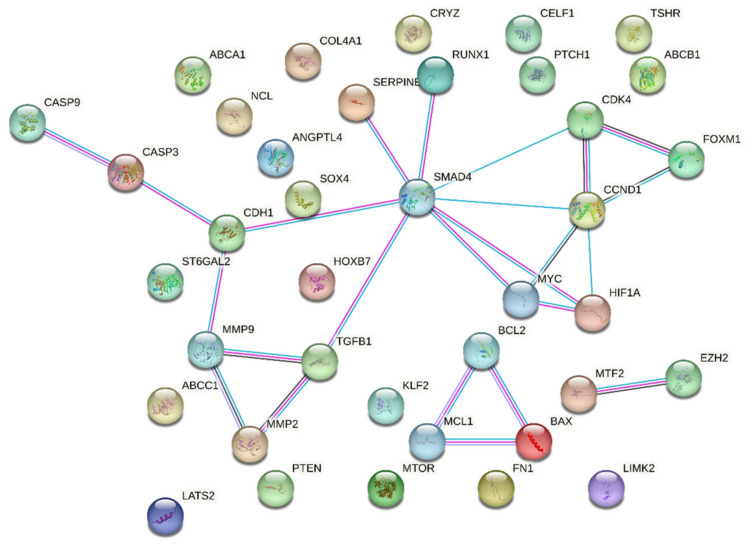
String output showing protein–protein interactions of 38 queried proteins. Colors legend: Red: inactivated, Green: activated, Blue: Indifferent.

Upon utilizing the STRING database with the set parameters and interaction sources, the resulting protein–protein interaction network revealed some intriguing statistics. The network comprised a total of 38 nodes representing individual proteins or genes; these nodes were interconnected through 108 edges signifying potential interactions between the proteins. On average, each node in the network demonstrated a degree of 5.68, indicating that on average each protein or gene interacts with approximately five to six other entities in the network. *SMAD4* and *CCND1* were leading hub nodes, with node degrees of 8 and 5, respectively. Upon further examining the intricacy of these interactions, the average local clustering coefficient was found to be 0.535 (Figure 4). 

This metric suggests a moderate tendency for the proteins to cluster together, forming interconnected groups within the network. Interestingly, when contrasting the observed data against the expected norm, the expected number of edges for such a network was only 18. This significant deviation from the expected values indicates that the compiled list of genes showcases a denser interaction than would typically be predicted for a random set of proteins of a similar size. This observation was statistically fortified with a PPI enrichment *p*-value of less than 1.0 × 10^−16^, confirming that the network contained a significantly higher number of interactions than expected.

Moreover, delving deeper into the local network clusters from STRING, several crucial biological processes and pathways surfaced, showcasing the potential roles of our genes of interest. Among the four emergent clusters, the cluster associated with “Apoptosis—Multiple Species and TRAIL Signaling” sheds light on the roles of proteins such as *BAX*, *CASP3*, *CASP9*, *MCL1*, and *BCL2*. Next, our attention was drawn to the “Bcl-2 Family and BH3-only Proteins Associate with and Inactivate Anti-apoptotic BCL-2 Members”. This cluster underscores the intricate interactions and regulatory mechanisms orchestrated by proteins such as *BAX*, *MCL1*, and *BCL2*. A third significant cluster, titled “Extracellular Matrix Organization”, unveils insights into the operational dynamics of proteins pivotal for the ECM, including *MMP2*, *TGFB1*, *FN1*, *MMP9*, and *COL4A1*. Concluding the list of notable clusters, the “Activation of Caspases through Apoptosome-mediated Cleavage” cluster highlights the indispensable roles played by *CASP3* and *CASP9* in the intricate dance of apoptosis. A comprehensive overview of the False Discovery Rate (FDR) values and the strength associated with these clusters is available in Table 2.

### 3.6. Ingenuity Pathway Analysis

**Figure 5:**
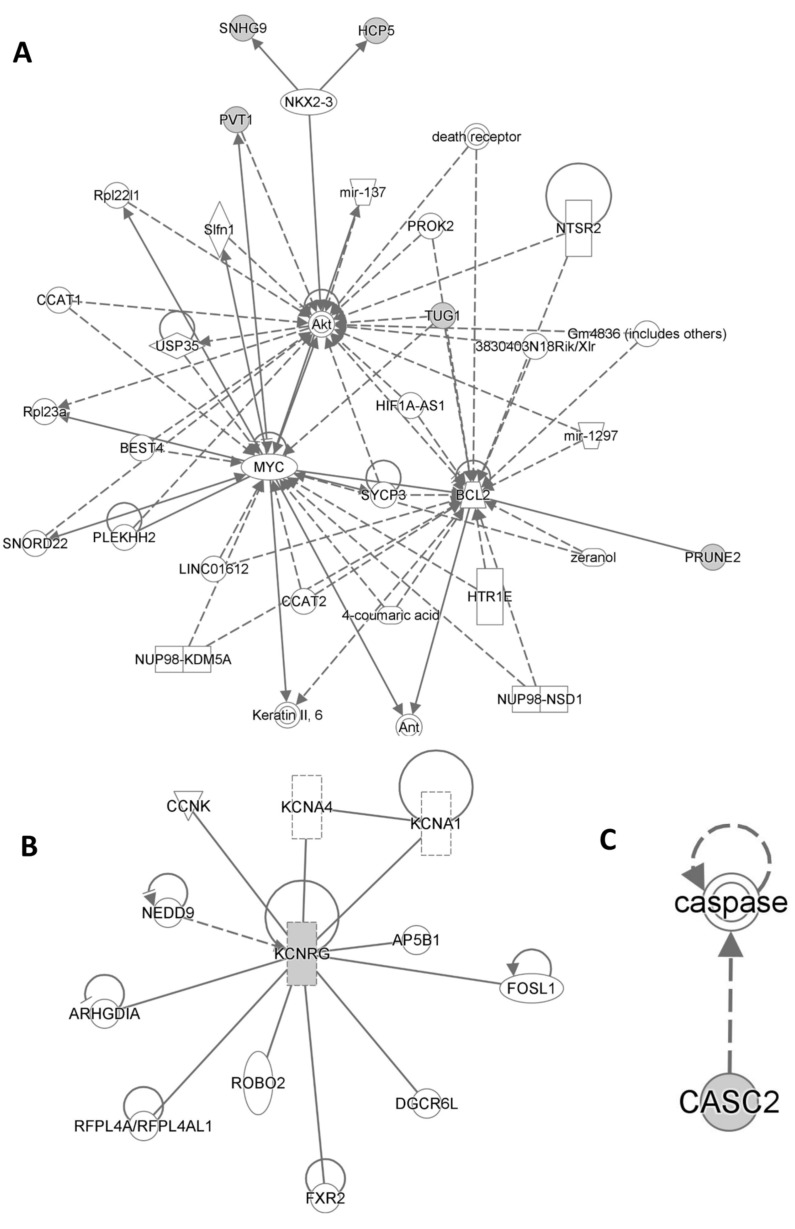
Statistically significant networks and pathways related to the lncRNA typical of HS patients from Ingenuity Pathway Analysis are shown in (**A**), (**B**), (**C**) respectively.

Ingenuity Pathway Analysis (IPA QIAGEN) evidenced that Cancer, Organismal Injury and Abnormalities, Cell Death and Survival, Organismal Injury and Abnormalities as well as Cellular Growth and Proliferation, Organ Development, Reproductive System Development and Function, and Tissue Development were included among statistically significant diseases and functions (Supplementary Tables S1 and S2). On the other hand, Cancer, Cell Death and Survival, Organismal Injury and Abnormalities, Cell Signaling, Cellular Development, Cellular Growth and Proliferation, Cardiovascular Disease, Neurological Disease, and Ophthalmic Disease were included among statistically significant networks (Figure 5). 

## 4. Discussion

Several important stages are hallmarks in the course of HS development, during which distinctive lncRNAs play an essential role. Particularly when deregulated, they disrupt the delicate balance between damaging and reparative processes, which in turn can exacerbate chronic inflammation and hinder wound healing and tissue renovation. lncRNAs contribute significantly to HS pathogenesis and progression through regulation of gene expression and molecular signaling pathways [30]. Despite the fact that some lncRNAs have been linked to several biological processes, their precise roles have not yet been fully studied in the setting of HS pathogenetic mechanisms.

As reported in the Results section, we pinpointed statistically significant differential methylation at CpG sites of lncRNA coding genes; *DLEU2*, *MESTIT1*, *CASC2*, *TUG1*, *KCNQ1DN*, *PSORS1C3*, *PCA3*, *DSCR8*, *RFPL1S*, and *PVT1* were hypomethylated, while *HAR1A*, *FAM66B*, *SNHG9*, *HCG9*, and *HCP5* were hypermethylated.

### 4.1. PCA3

Prostate Cancer Antigen 3 (*PCA3*) is a gene that has primarily been associated with prostate cancer; its expression is used as a diagnostic marker for the disease [31]. *PCA3* modulates prostate cancer (PCa) cell survival through modulating androgen receptor (AR) signaling [32]. AR is a nuclear hormone receptor that binds to androgens such as testosterone and dihydrotestosterone (DHT) and regulates the transcription of target genes involved in cell proliferation, differentiation, and survival. In HS, the overexpression and activation of AR have been implicated in the increased proliferation and abnormal differentiation of the hair follicles and apocrine glands in the affected areas, leading to the formation of inflammatory nodules and cysts [33]. 

### 4.2. DSCR8

*DSCR8* gene (Down syndrome critical region 8), also known as *MMA*-*1* (Malignant melanoma-associated protein 1), is highly expressed in uterine cancer and melanoma [34]. The prevalence of non-melanoma skin cancer risk is augmented in correlation with HS [35]. It may be possible that *DSCR8* regulates immune responses and inflammation, which in turn contribute to HS development. Moreover, many studies have reported an increased prevalence of Down syndrome in the HS population [36,37,38]. 

### 4.3. TUG1

*TUG1* gene has been implicated in various biological processes, including cell proliferation, apoptosis, and inflammation. Although the exact role of *TUG1* in apocrine glands is not clear, some studies suggest that it may be involved in regulating the function of these glands. *TUG1* is highly expressed in human apocrine sweat glands and its expression is regulated by androgen hormones. *TUG1* is upregulated in the apocrine sweat glands of patients with HS. *TUG1* is a regulator of the *NLRP3* inflammasome, a key mediator of inflammation in HS. Earlier reports indicate that *TUG1* is upregulated in HS lesional skin and that knockdown of *TUG1* can reduce the production of IL-1β, a proinflammatory cytokine produced by the *NLRP3* inflammasome.

### 4.4. HAR1A

HS and atopic dermatitis (AD) are both chronic inflammatory skin diseases [39]. AD, also known as eczema, produces dry, itchy, and red skin; the *HAR1A* gene is implicated in AD [40]. A strong clinical association between HS and AD has been previously reported [39], suggesting that HS may share a more common genetic landscape.

### 4.5. DLEU2

HS-related genes such as *DLEU2* [41], which regulates Sirtuins and mitochondrial respiratory chain complex IV, are associated with the regulation of mitochondrial function, indicating that mitochondrial dysfunction could play a central role in HS pathogenesis. *DLEU2* is involved in several types of cancer, including chronic lymphocytic leukemia and non-small-cell lung cancer. Loss of the *DLEU2* gene is associated with an increased risk of squamous cell carcinoma of the skin. Furthermore, *DLEU2* expression is significantly decreased in melanoma samples compared to normal skin. 

### 4.6. HCG9

Several studies have found an association between *HCG9* gene changes and bipolar disorder [42]. Although it is common for patients with HS to have psychiatric comorbidities, few studies have examined whether severe psychiatric disorders are associated with HS. However, a recent population-based study found that HS patients are more likely to suffer from bipolar disorder [43]. 

### 4.7. CASC2

*CASC2* (Cancer susceptibility 2) is a tumor suppressor gene that has been associated with several types of cancers, including endometrial, lung, gastric, and colorectal cancers [44]; however, there is limited research on its possible involvement in HS. HS seems to cause an increased risk of developing several types of cancer, including lung, gastric, and colorectal cancers, as well as earlier onset [35]. Additionally, CASC2 may inhibit the development of malignant melanoma by regulating miR-18a-5p/RUNX1 axis [45]. Both *CASC2* and *RUNX1* were hypomethylated in the present study. 

### 4.8. FAM66B

*FAM66B* gene changes have been reported to be involved in esophageal squamous cell carcinoma [46]. However, the association of such changes with HS has not been studied in depth yet.

### 4.9. KCNQ1DN

*KCNQ1DN* is an imprinted gene located between p57(KIP2) and *KvLQT1* (*KCNQ1*) in chr11p15.5 within the Wilms’ tumorigenesis (WT2) critical region, and is considered a candidate for involvement in WT. 

### 4.10. RFPL1S

The tumor suppressor gene *RFPL1S* may slow ovarian cancer progression by inhibiting IFN-β/STAT1 signaling [47]. 

### 4.11. SNHG9

The *SNHG9* gene has been implicated in several cancer types, including glioblastoma [48], pancreatic cancer [49], and non-small cell lung cancer (SCLC) [50], although its function in HS remains unknown. The majority of HS patients are either active or passive smokers; smoking is a known risk factor for lung cancer, including SCLC, and smoking rates are higher among subjects suffering from HS compared to the general population.

### 4.12. MESTIT1

*MESTIT1* is an imprinted gene preferentially expressed from the paternal allele [51]. *MESTIT1* has been reported as a strong candidate gene for Silver–Russell syndrome (SRS) in chr7q31 [52]. Approximately 7–10% of SRS cases have been reported with maternal uniparental disomy (UPD) of chromosome 7, a segmental maternal UPD (7) restricted to 7q31-other. The involvement of *MESTIT1* in HS pathophysiology is unknown at present. 

### 4.13. PSORS1C3

*PSORS1C3* is associated with the development of psoriasis, a common chronic immune-mediated inflammatory disease of the skin [53]. Psoriasis is one of the critical comorbidities associated with HS [54].

### 4.14. PVT1

*PVT1* has been linked to a variety of cancers, including gastric cancer, breast cancer, ovarian cancer, and skin cancer [55]. *PVT1* functions as an oncogene by inhibiting cancer cell apoptosis, promoting cell proliferation, and affecting tumor invasion and metastasis generation [56]. When downregulated, *PVT1* is thought to be involved in the regulation of inflammation, as it reduces the expression of inflammatory cytokines such as *TNF*-*α*, *IL*-*1β*, *IL*-*6*, *IL*-*10*, *IL*-*17*, and *IFN*-*γ* [57], suggesting that *PVT1* may contribute to the inflammatory response in HS.

### 4.15. HCP5

*HCP5* (HLA complex P5) is a gene that encodes a protein involved in the immune response. Variants of the *HCP5* gene are associated with an increased risk of developing Stevens–Johnson syndrome (SJS) and toxic epidermal necrolysis (TEN), which are severe and potentially life-threatening cutaneous drug reactions [58,59]. Sorafenib treatment has been found to cause adverse cutaneous reactions and side effects in HS patients [60]. There is evidence that hypermethylation of *HCP5* relates to obesity and BMI in Africans [61]. Obesity and overweight are the most common comorbid conditions associated with HS.

### 4.16. Protein–Protein Interactions

PPIs and lncRNA expression are two important aspects of molecular biology that play crucial roles in cellular processes and gene regulation. LncRNAs regulate gene expression by interacting with intracellular molecules such as DNA, RNA, and proteins. They can bind DNA to influence protein recruitment, control mRNA stability and translation, and form ribonucleoprotein complexes for diverse cellular functions.

In the present study, we looked for lncRNA targets through database mining and retrieved 38 lncRNA target genes. These genes were further subjected to STRING protein–protein interaction network analysis to search for interactions among them. We found two hub nodes showing maximum interaction among the group components, namely, *SMAD4* (target of lncRNA *PVT1*) and *CCND1* (target of lncRNA *TUG1*). Increased risk for SCC or adenocarcinoma has been reported in HS patients with abnormal *CCND1* expression, possibly due to chronic inflammation [62]. Results from a GWAS study from PIONEER I and II clinical trial participants have highlighted a pathway involving *BCL2* in response to adalimumab, the only FDA approved drug for HS treatment [63]. In addition, among the 38 gene result lncRNA targets, *SERPINE1*, *MTOR*, and *MMP9* have been reported to be suitably druggable [64]. 

Recent genome-wide association studies (GWASs) have been performed for HS, which have identified and replicated significant HS-associated risk loci. Of particular interest were the lead variants rs10512572 (p = 2.3 × 10^−11^) and rs17090189 (p = 2.1 × 10^−8^) near the SOX9 and KLF5 genes, respectively; variants at these loci resulted in enhanced regulatory elements detected in skin tissue [65].

Furthermore, the results of a recent cross-sectional genotype–phenotype study performed in a Maltese patient cohort propose that monogenic variation in NCSTN, one of the genes that code for proteins of the γ secretase complex, is associated with HS in a subset of patients with a different nonconforming phenotype. In addition, carriers of the NCSTN:c.671_682del variant were more likely to require adalimumab treatment [66].

### 4.17. Identifying Druggable Targets

Identifying druggable targets typically involves extensive research into the biological pathways, functions, and potential therapeutic applications of the studied molecules. LncRNAs presents unique challenges compared to protein-coding genes. Traditional small molecule drugs, which primarily target proteins, are not applicable for lncRNAs, as they lack a protein-coding function and instead function as regulators of gene expression. While several emerging strategies to potentially target lncRNAs for therapeutic purposes are currently being researched and developed in preclinical testing, these approaches are relatively new, albeit promising. They include antisense oligonucleotides (ASOs), small interfering RNAs (siRNAs), CRISPR-Cas9-based therapies, and RNA interference-based therapies.

### 4.18. Limitations

The current findings are limited by a lack of experimental in vivo and in vitro validation. Future experimental studies are needed in order to corroborate the expression and function of the identified genes at the protein level, determine whether these changes in pathway activation are associated with HS disease activity, and identify which molecules can be used to develop targeted therapies for HS. In addition, the validity of the lncRNAs needs to be verified in a large HS population study. While various lncRNAs are candidate central regulators in inflammatory signaling pathways, only a few of them have been pinpointed in HS. The shared mechanisms of HS and other inflammatory diseases imply a similar role of lncRNAs guiding the onset of HS. It would be informative to uncover the functions of these lncRNAs in the context of HS.

Furthermore, as evidenced by IPA, changes in genes encoding lncRNAs in the context of HS suggest enrichment of pathways linked to carcinogenesis or cellular processes essential for upholding tissue homeostasis, such as cell death/survival and cell growth/proliferation.

## 5. Conclusions

LncRNAs are excellent targets to (a) further understand HS pathogenesis, (b) decipher the molecular mechanisms involved in HS-driven inflammation, and (c) train the development of new effective targeted therapies. A recent systematic review aiming to classify all recognized HS biomarkers evaluated the results of randomized clinical trials, uncontrolled clinical trials, cohort studies, case-control studies, and other observational studies published up until 31 December 2020 without exclusion criteria related to patient age, sex, race or ethnicity, or language. Among the 48 acknowledged biomarkers, one diagnostic biomarkers (serum IL-2R), one monitoring biomarkers (dermal Doppler vascularity), and two predictive biomarkers (epithelialized tunnels and positive family history of HS) were found with high GRADE ratings. However, none of them could be recommended for routine use in the clinical setting [67]. 

HS ensues after puberty; thus, hormones could play an important role in its pathogenesis. However, randomized controlled trials and experimental studies investigating the influence of hormones in HS are lacking. Currently, no relationship among deregulated lncRNAs and hormone secretion has been found. On the other hand, the role of sex hormones such as androgens and estrogens, adipokines, thyroid hormones, insulin resistance, and alteration of the insulin-driven immune–metabolic axis is under intense evaluation in HS pathophysiology [68]. As proposed in our study, blood-based biomarkers could provide a less invasive and more efficient tool for detecting skin diseases, and specifically HS, particularly in the early stages when treatment may be more effective. Although more research is needed in order to fully understand the role of lncRNAs in skin diseases and develop reliable biomarkers, the increasing interest and investment in this area suggests that progress is being made. Understanding the role played by lncRNAs will likely lead to novel mechanistic insights into HS pathogenesis, leading to new therapeutic options. 

## Figures and Tables

**Table 1:** Significantly differentially methylated CpG sites of lncRNA associated with HS. Differentially methylated CpG sites and regions related to HS with Target ID, Gene ID, chromosome location, % methylation change, and FDR *p*-value for each CpG loci are provided. CpG sites with a significant FDR *p*-value indicating methylation status and ROC AUC > 0.75 appear to be potential diagnostic biomarkers for HS.

Target ID	Gene Name	Chromosome Locus	*p*-Val	FDR *p*-Val	% Methylation Cases	% Methylation Control	% Methlation Change	AUC	CI_Lower	CI_Upper
cg06254801	PCA3	9q21.2	1.8576 × 10^−8^	0.01606824	64.86	73.09	−8.23	0.95	0.89	1.00
cg07064066	DSCR8	21q22.13	1.56 × 10^−8^	0.01346011	74.59	81.52	−6.93	0.91	0.83	1.00
cg04270033	TUG1	22q12.2	5.48373 × 10^−11^	4.74343 × 10^−5^	67.91	76.96	−9.05	0.91	0.83	1.00
cg08221811	HAR1A	20q13.33	4.73141 × 10^−8^	0.040926711	9.68	5.19	4.49	0.86	0.75	0.97
cg17948986	DLEU2	13q14.2	2.2571 × 10^−16^	1.9524 × 10^−10^	60.64	72.88	−12.24	0.85	0.74	0.96
cg08072458	HCG9	6p22.1	7.93312 × 10^−11^	6.86215 × 10^−5^	12.15	6.41	5.73	0.85	0.73	0.96
cg17374433	CASC2	10q26.11	1.80494 × 10^−10^	0.000156127	60.88	70.62	−9.73	0.84	0.73	0.96
cg02330432	FAM66B	8p23.1	4.01146 × 10^−8^	0.034699166	9.66	5.15	4.51	0.83	0.71	0.95
cg10503232	KCNQ1DN	11p15.5	1.26385 × 10^−8^	0.010932294	63.61	72.08	−8.47	0.83	0.71	0.95
cg08653574	RFPL1S	22q12.2	1.28161 × 10^−12^	1.10859 × 10^−6^	83.55	90.05	−6.50	0.80	0.68	0.93
cg21871735	SNHG9	16p13.3	2.2517 × 10^−10^	0.000194772	8.97	4.00	4.96	0.80	0.68	0.93
cg10473158	MESTIT1	7q32.2	1.29239 × 10^−10^	0.000111792	56.09	66.36	−10.28	0.80	0.67	0.92
cg11040238	PSORS1C3	6p21.33	1.14754 × 10^−8^	0.009926202	65.37	73.65	−8.29	0.79	0.66	0.92
cg08588859	PVT1	8q24.21	2.07709 × 10^−10^	0.000179668	90.18	94.53	−4.35	0.77	0.64	0.91
cg08099293	HCP5	6p21.33	1.10616 × 10^−14^	9.56827 × 10^−9^	12.88	6.10	6.78	0.75	0.62	0.89

**Table 2:** Local Network Clusters from STRING.

Cluster ID	Description	Observed Gene Count	Background Gene Count	Strength	FDR	Matching Proteins (Labels)
CL:16888	Apoptosis—Multiple Species, and TRAIL signaling	5	35	1.87	5.77 × 10^−5^	BAX, CASP3, CASP9, MCL1, BCL2
CL:16889	Bcl-2 family, and BH3-only proteins associate with BCL-2 members	3	18	1.94	0.0078	BAX, MCL1, BCL2
CL:19457	Extracellular matrix organization	5	180	1.16	0.0204	MMP2, TGFB1, FN1, MMP9, COL4A1
CL:16926	Activation of caspases through apoptosome-mediated cleavage	2	5	2.32	0.0492	CASP3, CASP9

## Data Availability

The published article and its Supplementary Materials contain all the data generated during this study.

## Supplementary Materials

The following supporting information can be downloaded at: https://www.mdpi.com/article/10.3390/jcm13103016/s1, Table S1: Diseases and Functions associated to HS-related lncRNAs; Table S2: Networks associated to HS-related lncRNAs.
