# Supplementary material for: Deregulated Long Non-Coding RNAs (lncRNA) as Promising Biomarkers in Hidradenitis Suppurativa
Source: J Clin Med. 2024 May 20;13(10):3016. doi: 10.3390/jcm13103016 (PMC11121919; doi:10.3390/jcm13103016)
Supplement: Supplementary file 1 [file jcm-13-03016-s001.zip › jcm-2966405-supplementary.pdf]

Journal of Clinical Medicine

MDPI

Supplementary Materials

Supplementary Table S1. Diseases and Functions associated to HS-related lncRNAs.

Categories	Functions	Diseases or Functions Annotation	p-value	B-H p-value	Molecules	# Molecules
Cancer,Organismal Injury and Abnormalities,Renal and Urological Disease	renal clear cell cancer	Renal clear cell adenocarcinoma	0,000183	0,0168	CASC2,HCP5,PRUNE2,PSORS1C3,PVT1	5
Cancer,Organismal Injury and Abnormalities	malignant neoplasm of retroperitoneum	Malignant neoplasm of retroperitoneum	0,000268	0,0168	CASC2,HCP5,KCNRG,PRUNE2,PSORS1C3,PVT1,TUG1	7
Cancer,Organismal Injury and Abnormalities	extraadrenal retroperitoneal tumor	Extraadrenal retroperitoneal tumor	0,000486	0,0168	CASC2,HCP5,KCNRG,PRUNE2,PSORS1C3,PVT1,TUG1	7
Cell Death and Survival,Organismal Injury and Abnormalities	apoptosis	Apoptosis of squamous cell carcinoma cell lines	0,000724	0,0168	PVT1,TUG1	2
Endocrine System Disorders,Gastrointestinal Disease,Immunological Disease,Metabolic Disease,Organismal Injury and Abnormalities	insulin-dependent diabetes mellitus	Insulin-dependent diabetes mellitus	0,000983	0,0168	HCG9,HCP5,PRUNE2	3
Cellular Growth and Proliferation,Organ Development,Reproductive System Development and Function,Tissue Development	proliferation	Proliferation of endometrial epithelial cell	0,000998	0,0168	TUG1	1
Cellular Movement	invasion	Invasion by endometrial epithelial cell	0,000998	0,0168	TUG1	1
Cellular Movement	migration	Migration of endometrial epithelial cell	0,000998	0,0168	TUG1	1
Cancer,Organismal Injury and Abnormalities,Tissue Morphology,Tumor Morphology	volume	Volume of tumor	0,00131	0,0168	PVT1,TUG1	2
Cell Cycle	Gap 0-Gap 1 phase	Arrest in Gap 0-Gap 1 phase of skin cancer cell lines	0,00133	0,0168	PVT1	1
Cellular Movement	migration	Migration of squamous cell carcinoma cell lines	0,00145	0,0168	PVT1,TUG1	2
Cell Death and Survival,Organismal Injury and	apoptosis	Apoptosis of ovarian	0,0	0,01	PVT1,TUG1	2

Abnormalities		cancer cell lines	016	68		
Hematological Disease,Immunological Disease,Metabolic Disease,Organismal Injury and Abnormalities	IgA deficiency	IgA deficiency	0,00166	0,0168	PVT1	1
Cellular Development,Cellular Growth and Proliferation	colony formation	Colony formation of skin cancer cell lines	0,00166	0,0168	PVT1	1
Cancer,Organismal Injury and Abnormalities,Respiratory Disease	laryngeal squamous cell carcinoma	TNM stage T3-4 laryngeal squamous cell carcinoma	0,00266	0,025	TUG1	1
Cellular Development,Cellular Growth and Proliferation	cell proliferation	Cell proliferation of ovarian cancer cell lines	0,00303	0,0261	PVT1,TUG1	2
Cell Death and Survival	cell viability	Cell viability of skin cancer cell lines	0,00332	0,0261	PVT1	1
Immunological Disease,Organismal Injury and Abnormalities	systemic autoimmune syndrome	Systemic autoimmune syndrome	0,00333	0,0261	HCG9,HCP5,PRUNE2,PVT1	4
Cell Cycle	Gap 0-Gap 1 phase	Arrest in Gap 0-Gap 1 phase of squamous cell carcinoma cell lines	0,00366	0,0271	PVT1	1
Cancer,Organismal Injury and Abnormalities	development	Development of subcutaneous tumor	0,00432	0,0298	PVT1	1
Cancer,Organismal Injury and Abnormalities	metastasis	Metastasis of kidney cancer cell lines	0,00465	0,0298	PVT1	1
Tissue Morphology	quantity	Quantity of squamous cell carcinoma cell lines	0,00465	0,0298	TUG1	1
Dermatological Diseases and Conditions,Immunological Disease,Organismal Injury and Abnormalities	vitiligo vulgaris	Vitiligo vulgaris	0,00697	0,0427	HCG9	1
Cellular Development,Cellular Growth and Proliferation	colony formation	Colony formation of squamous cell carcinoma cell lines	0,0073	0,0429	PVT1	1
Cancer	sphere formation	Sphere formation of colorectal cancer cell lines	0,00796	0,0432	PVT1	1
Cell Death and Survival,Organismal Injury and Abnormalities	apoptosis	Apoptosis of skin cancer cell lines	0,0079	0,0432	PVT1	1

			6			
Gastrointestinal Disease,Hepatic System Disease,Organismal Injury and Abnormalities	drug-induced liver disease	Drug-induced liver disease	0,0 089 5	0,04 68	HCP5	1
Endocrine System Disorders,Gastrointestinal Disease,Metabolic Disease,Organismal Injury and Abnormalities	diabetes mellitus	Diabetes mellitus	0,0 096 2	0,04 84	HCG9,HCP5,PRUNE2, PVT1	4
Cellular Movement	migration	Migration of skin cancer cell lines	0,0 109	0,05 32	PVT1	1
Immunological Disease,Organismal Injury and Abnormalities	immunodeficien cy	Immunodeficiency	0,0 118	0,05 56	HCP5,PVT1	2
Cellular Assembly and Organization,Cellular Function and Maintenance	binding	Binding of microtubules	0,0 132	0,06 02	PRUNE2	1
Cancer,Hematological Disease,Immunological Disease,Organismal Injury and Abnormalities	nodular sclerosis Hodgkin disease	Nodular sclerosis classical Hodgkin lymphoma	0,0 139	0,06 12	PRUNE2	1
Cell Death and Survival	cell viability	Cell viability of squamous cell carcinoma cell lines	0,0 152	0,06 5	PVT1	1
Cancer,Organismal Injury and Abnormalities,Reproductive System Disease	metaplastic breast cancer	Metaplastic breast carcinoma	0,0 175	0,07 26	PRUNE2	1
Immunological Disease,Infectious Diseases,Organismal Injury and Abnormalities	acquired immunodeficien cy syndrome	Acquired immunodeficiency syndrome	0,0 191	0,07 63	HCP5	1
Cellular Development,Cellular Growth and Proliferation	proliferation	Proliferation of endometrial cancer cell lines	0,0 195	0,07 63	CASC2	1
Cellular Movement	invasion	Invasion of carcinoma cell lines	0,0 21	0,07 95	PVT1,TUG1	2
Cancer,Endocrine System Disorders,Organismal Injury and Abnormalities,Reproductive System Disease	carcinoma in situ of testis	Carcinoma in situ of testis	0,0 214	0,07 95	PRUNE2	1
Organismal Injury and Abnormalities,Reproductive System Disease	adenomyosis	Adenomyosis	0,0 234	0,08 46	TUG1	1
Cellular Movement	migration	Migration of kidney cancer cell lines	0,0 253	0,08 93	PVT1	1
Cellular Development,Cellular Growth and Proliferation	cell proliferation	Cell proliferation of tumor cell lines	0,0 274	0,09 42	CASC2,KCNRG,PVT1, TUG1	4
Cellular Development,Cellular Growth and Proliferation	proliferation	Proliferation of myeloma cell lines	0,0 325	0,10 9	KCNRG	1
Cancer,Organismal Injury and Abnormalities	metastasis	Metastasis of colorectal cancer cell lines	0,0 335	0,11	PVT1	1
Cell Death and Survival	cell viability	Cell viability of pancreatic cancer cell lines	0,0 351	0,11 2	TUG1	1

Cancer,Organismal Injury and Abnormalities,Tissue Morphology,Tumor Morphology	mass	Mass of tumor	0,0367	0,113	TUG1	1
Cell Death and Survival,Organismal Injury and Abnormalities	apoptosis	Apoptosis of myeloma cell lines	0,037	0,113	KCNRG	1
Cellular Movement	invasion	Invasion of colorectal cancer cell lines	0,038	0,114	PVT1	1
Immunological Disease,Inflammatory Disease,Organismal Injury and Abnormalities	immune mediated inflammatory disease	Immune mediated inflammatory disease	0,0388	0,114	HCG9,PRUNE2,PVT1	3
Cell Death and Survival,Organismal Injury and Abnormalities	apoptosis	Apoptosis of tumor cell lines	0,0409	0,118	KCNRG,PVT1,TUG1	3
Cancer,Organismal Injury and Abnormalities	upper abdominal cancer	Upper abdominal cancer	0,0421	0,119	CASC2,HCP5,KCNRG,PRUNE2,PSORS1C3,PVT1,TUG1	7
Cellular Movement	migration	Migration of pancreatic cancer cell lines	0,0453	0,121	TUG1	1
Cell Death and Survival,Organismal Injury and Abnormalities	apoptosis	Apoptosis of vascular endothelial cells	0,0453	0,121	TUG1	1
Infectious Diseases,Organismal Injury and Abnormalities	HIV infection	HIV infection	0,0455	0,121	HCP5,PVT1	2

Supplementary Table S2. Networks associated to HS-related lncRNAs.

ID	Molecules in Network	Score	Focus Molecules	Top Diseases and Functions				
1	3830403N18Rik/Xlr,4-coumaric acid,Akt,Ant,BCL2,BEST4,CCAT1,CCAT2,death receptor,Gm4836 (includes others),HCP5,HIF1A-AS1,HTR1E,Keratin II, 6,LINC01612,mir-1297,mir-137,MYC,NKX2-3,NTSR2,NUP98-KDM5A,NUP98-NSD1,PLEKHH2,PROK2,PRUNE2,PVT1,Rpl22l1,Rpl23a,Slfn1,SNHG9,SNORD22,SYCP3,TUG1,USP35,zeranol	14	5	[Cancer, Cell Death and Survival, Organismal Injury and Abnormalities]				
2	CASC2,caspase	3	1	[Cell Signaling, Cellular Development, Cellular Growth and Proliferation]				
3	AP5B1,ARHGDIA,CCNK,DGCR6L,FOSL1,FXR2,KCNA1,KCNA4,KCNRG,NEDD9,RFPL4A/RFPL4AL1,ROBO2	3	1	[Cardiovascular Disease, Neurological Disease, Ophthalmic Disease]				
